# Urine IP-10 as a biomarker of therapeutic response in patients with active pulmonary tuberculosis

**DOI:** 10.1186/s12879-018-3144-3

**Published:** 2018-05-29

**Authors:** Song Yee Kim, Jungho Kim, Deok Ryun Kim, Young Ae Kang, Sungyoung Bong, Jonghee Lee, Suyeon Kim, Nam Suk Lee, Bora Sim, Sang-Nae Cho, Young Sam Kim, Hyejon Lee

**Affiliations:** 1 Division of Pulmonary, Department of Internal Medicine, Institute of Chest Diseases, Severance Hospital, Yonsei University College of Medicine, Seoul, 03722 Republic of Korea; 2 Clinical Vaccine Research Section, International Tuberculosis Research Center, Seoul, 03722 Republic of Korea; 3 Department of Biomedical Laboratory Science, College of Health Sciences, Yonsei University, Wonju, 26493 Republic of Korea; 4 Development and Delivery Unit, International Vaccine Institute, Seoul, 08826 Republic of Korea; 5 Department of Microbiology and Institute of Immunology and Immunological Disease, Yonsei University College of Medicine, Seoul, 03722 Republic of Korea

**Keywords:** Tuberculosis, Urine IP-10, Biomarker, Monitoring

## Abstract

**Background:**

Prior to clinical trials of new TB drugs or therapeutic vaccines, it is necessary to develop monitoring tools to predict treatment outcomes in TB patients. Urine interferon gamma inducible protein 10 (IP-10) is a potential biomarker of treatment response in chronic hepatitis C virus infection and lung diseases, including tuberculosis. In this study, we assessed IP-10 levels in urine samples from patients with active TB at diagnosis, during treatment, and at completion, and compared these with levels in serum samples collected in parallel from matched patients to determine whether urine IP-10 can be used to monitor treatment response in patients with active TB.

**Methods:**

IP-10 was measured using enzyme-linked immunosorbent assays in urine and serum samples collected concomitantly from 23 patients with active TB and 21 healthy adults (44 total individuals). The Mann-Whitney *U* test and Wilcoxon matched-pairs signed rank test were used for comparisons among healthy controls and patients at three time points, and LOESS regression was used for longitudinal data.

**Results:**

The levels of IP-10 in urine increased significantly after 2 months of treatment (*P* = 0.0163), but decreased by the completion of treatment (*P* = 0.0035). Serum IP-10 levels exhibited a similar trend, but did not increase significantly after 2 months of treatment in patients with active TB.

**Conclusions:**

Unstimulated IP-10 in urine can be used as a biomarker to monitor treatment response in patients with active pulmonary TB.

## Background

Tuberculosis (TB) remains a major infectious cause of mortality and morbidity, with approximately 8.6 million new cases and 1.3 million deaths per year [1]. In addition, there is concern over increasing rates of disease recurrence (including relapse and reinfection) after anti-TB drug therapy and an increasing trend of multidrug- and extensively drug-resistant (M/XDR) TB worldwide. Progress has been made in the control of tuberculosis, as advocated by the World Health Organization (WHO) [2].

For the development of novel vaccines and drugs against TB, a reliable surrogate marker of risk and protection against TB is essential. In particular, prior to clinical trials of new therapeutic vaccines to prevent TB recurrence and to shorten the duration of therapy, effective monitoring tools are needed to measure treatment responses to standard drug therapies in parallel with therapeutic vaccinations [3, 4].

Generally, sputum culture conversion after 2 months of anti-TB drug treatment has been used as a predictive indicator of drug treatment response in patients [5–9]. However, culture methods are not applicable in patients with extrapulmonary TB and culture-negative pulmonary TB, and laboratories capable of sputum culture are often lacking in resource-limited settings [10–12]. To accommodate these shortcomings, efforts have been made to identify an alternative biomarker for anti-TB treatment efficacy [4, 13, 14].

Interferon gamma (IFN-γ)-inducible protein (IP-10) is a pro-inflammatory chemokine secreted by antigen-presenting cells that transport activated T lymphocytes to sites of inflammation [15, 16]. IP-10 levels were found to be higher in the unstimulated serum, plasma, and urine of patients with active TB than those in subjects without active TB, and IP-10 levels decrease significantly after the completion of anti-TB treatment [15, 17–20]. Among all types of clinical specimens in which IP-10 has been detected, urine specimens are the most convenient and non-invasive; specimens can be obtained from children and elderly patients, even in resource-limited settings, as these samples do not require special equipment or skilful medical personnel for collection. In addition, urine specimens present lower handling risks compared to other specimens, such as blood, sputum, and microbial isolates from patients [20].

The aim of this study was to test the hypothesis that levels of urine IP-10 change over the course of anti-TB drug therapy in patients with active TB from South Korea, an area with an intermediate burden of TB and low burden of human immunodeficiency virus infection and acquired immune deficiency syndrome (HIV/AIDS).

## Methods

### Study population and setting

Clinical information and specimens were collected from participants enrolled in two clinical studies from November 2010 to March 2012 and from May 2015 to June 2016. The Institutional Ethics Committee of Yonsei University Severance Hospital approved this study (approval #4-2010-0527 and #4-2014-1108). All study participants provided written informed consent to provide specimens for the identification of TB biomarkers.

Study participants were classified as follows.

1. *Active pulmonary TB patients* (aged 20 years and over): Patients with active pulmonary TB were diagnosed based on culture and/or pathology. Cases of clinical active pulmonary TB, which was defined as negative mycobacterial culture findings, but favourable clinical and radiological responses to anti-TB medication, were also included. Patients were sub-divided into the following three groups based on risk factors for relapse, including the presence of (i) a cavity lesion on a chest X-ray or chest CT and (ii) a positive sputum culture after 2 months of anti-TB treatment as follows:

- *Low-risk group*: TB patients who had neither risk factor (i) nor (ii);
- *Moderate group*: TB patients who had either risk factor (i) or (ii);
- *High-risk group*: TB patients who had both risk factors (i) and (ii).

2) *Healthy controls* (aged 20 years and over): Healthy controls fulfilled the following inclusion criteria: (i) no history of tuberculosis, (ii) no suggestive symptoms of tuberculosis, (iii) no recent contact with patients with infectious tuberculosis, and (iv) negative results according to the QuantiFERON^®^-TB Gold In-Tube Test (QFT-GIT).

None of the participants exhibited HIV infection; a chronic comorbidity, such as diabetes mellitus, chronic renal failure, malignant tumour, or chronic liver disease; immunosuppressive status; or acute infections.

### IFN-γ measurement

A QFT-GIT assay was conducted for all patients before treatment (0 months, T0), at 2 months (T2), and at 6–9 months after the treatment was completed (T6).

The QFT-GIT assay (Qiagen, Hilden, Germany) was performed according to the manufacturer’s instructions. Briefly, 1 ml of whole blood was collected in each of three tubes pre-coated with *Mycobacterium tuberculosis*-specific peptides (ESAT-6, CFP-10, and TB7.7) or mitogen (positive control) and incubated for 16–24 h at 37 °C. For negative controls, whole blood was placed in tubes that were not pre-coated. The plasma supernatant was harvested by centrifugation at 3000×*g* for 15 min and stored at − 80 °C. The level of IFN-γ was determined using the QFT Enzyme-linked Immunosorbent Assay (ELISA) Kit. The results were interpreted using QFT-GIT software version 2.62, provided by the manufacturer.

### IP-10 measurement

Serum and urine samples were collected to measure serial IP-10 levels from all patients at T0, T2, and T6. IP-10 levels in urine and serum were concomitantly measured in duplicate using a commercial IP-10 ELISA Kit (R&D Systems, Minneapolis, MN, USA) in accordance with the manufacturer’s instructions. The test results were interpreted using SoftMax version 5.4.1 (Molecular Devices, LLC, Sunnyvale, CA, USA). In addition, using the test results available from routine hospital analyses, the urine IP-10 level (in pg/ml) was normalized against the serum creatinine level (in mg/ml) for each patient at each visit.

### Statistical analysis

All statistical analyses were performed using GraphPad Prism 6 software (GraphPad Software, La Jolla, CA, USA) and IBM SPSS software version 21.0 (IBM Corp., Armonk, NY, USA). Medians and interquartile range (IQR) were measured for continuous variables. The Mann–Whitney *U*-test and Wilcoxon matched-pairs signed rank test were used for comparisons among study groups and pairwise comparisons. A longitudinal smoothed trend line for the data (not based on any distributional assumption) was estimated using a nonparametric method called LOESS (locally weighted scatterplot smoothing). A 95% confidence limit was estimated, and similarity patterns by risk group were graphically examined. All *P*-values were two-sided, and *P* < 0.05 was considered statistically significant.

## Results

### Characteristics of study participants

We enrolled 23 patients with active TB and 21 healthy controls. The patients with active TB had a median age of 27 (range, 22–66), and the healthy controls had a median age of 25 (range, 20–33). Bacille Calmette-Guérin (BCG) scars were present in 65.2% of patients with active TB and 57.1% of healthy controls during inspection at the site. None of the enrolled patients were acid-fast bacillus smear-positive, 19 (82.6%) were culture-positive, and 8 (34.8%) had a cavity on a chest X-ray or computed tomography (CT) (Table 1).

**Table 1:** Characteristics of subjects enrolled in the study

		TB Patients (*n* = 23)	Healthy Controls (*n* = 21)
Median Age (range)		27 (22–66)	25 (20–33)
Male, n (%)		16 (59.3%)	13 (62.0%)
Presence of BCG^a^ scars		15 (65.2%)	12 (57.1%)
PTB^b^ diagnosis	AFB^c^ smear, positive	0 (0.0%)	0 (0.0%)
	AFB^c^ culture, positive	19 (82.6%)	0 (0.0%)
	Cavity on Chest X-ray or CT^d^	8 (34.8%)	0 (0.0%)
Risk group for relapse	High-risk	0 (0.0%)	0 (0.0%)
	Moderate-risk	8 (34.8%)	0 (0.0%)
	Low-risk	15 (65.2%)	0 (0.0%)

^a^*BCG* Bacille Calmette-Guérin, ^b^ *PTB* Pulmonary tuberculosis, ^c^ *AFB* Acid-fast bacillus; ^d^ *CT* Computed tomography. Risk factors for relapse: i) the presence of a cavity lesion on chest X-ray or chest CT, and ii) the result of a positive sputum culture after 2 months of anti-TB treatment. The high-risk group was defined when patients had both risk factors i) and ii). The moderate-risk group was defined when patients had either risk factor i) or ii), and the low-risk group had neither risk factor

### Comparison of serum and urine IP-10 levels between healthy controls and patients with TB at diagnosis

**Fig. 1:**
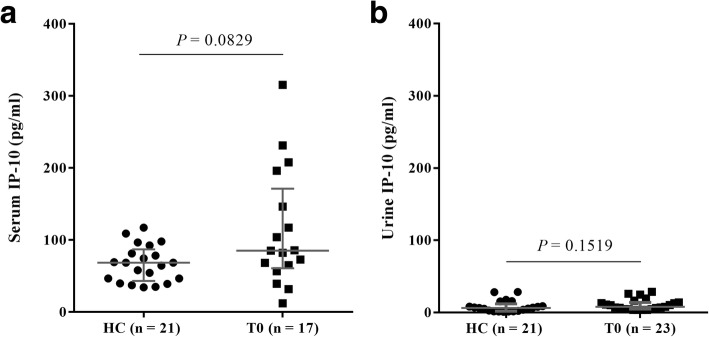
Serum and urine IP-10 levels in patients with TB at diagnosis and healthy controls. IP-10 levels were measured in the serum (**a**) and urine (**b**) of each TB patient at diagnosis (T0) and each healthy control (HC). We only obtained serum samples from 17 patients at T0 out of 23 patients. The data are shown as median with interquartile range; statistical analyses were performed using the Mann–Whitney *U*-test

We assessed whether the levels of IP-10 in serum and urine samples differed between TB patients at the time of diagnosis and healthy controls. Serum IP-10 levels did not differ significantly between TB patients at diagnosis (median, 85.37 pg/ml; IQR, 60.92-171.30) and healthy controls (median, 68.62 pg/ml; IQR, 43.19-87.01), although the levels in patients were slightly higher than those in the healthy controls (*P* = 0.0829; Fig. 1a). In addition, urine IP-10 levels in healthy controls (median, 6.49 pg/ml; IQR, 2.02-12.11) were not different from those in the TB patients at the time of diagnosis (median, 7.89 pg/ml; IQR, 4.86-13.97; Fig. 1b).

### Comparison of serum and urine IP-10 levels at TB diagnosis (T0), during treatment (T2), and after the completion of therapy (T6)

**Fig. 2:**
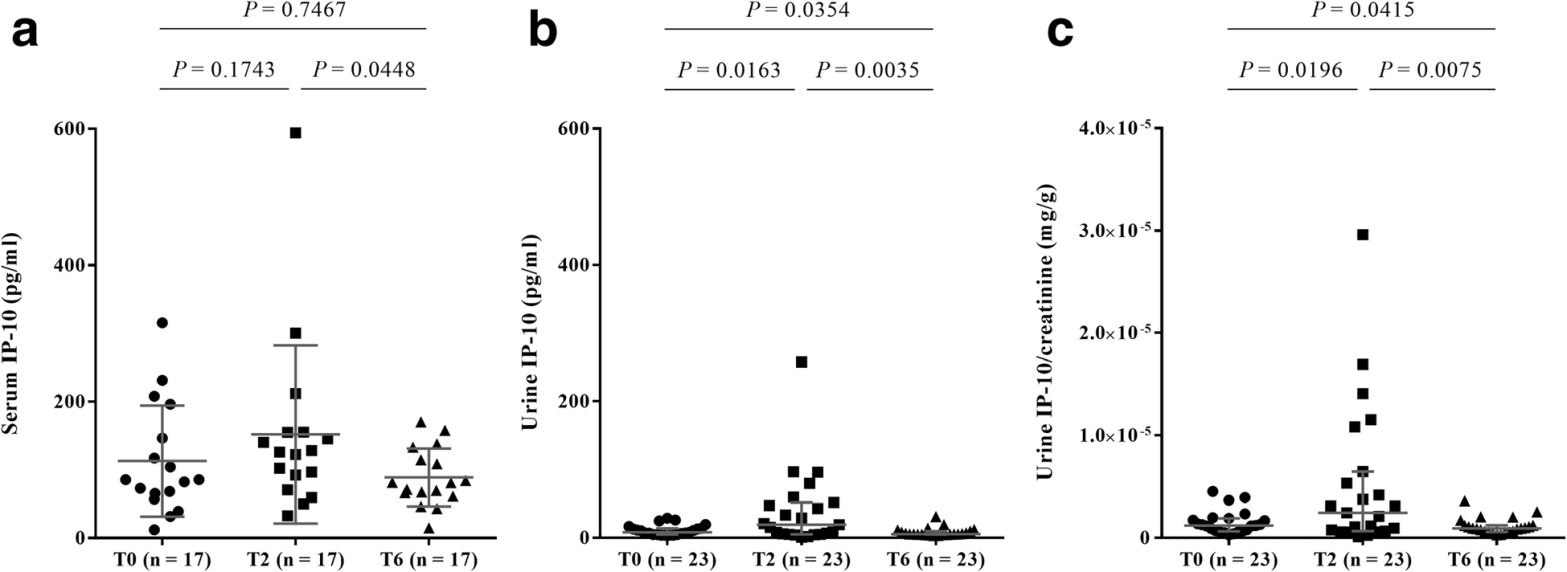
Serum and urine IP-10 levels in patients with TB before, during, and after treatment. Serial IP-10 levels in the (**a**) serum and (**b**) urine of patients with TB collected at the time of diagnosis (T0), after 2 months of therapy (T2), and after the completion of therapy (T6). **c** Serial IP-10 levels in urine normalized against serum creatinine levels in patients. The data are shown as median with interquartile range; statistical analyses were performed using the Wilcoxon matched-pairs signed rank test

We further analysed whether the serum and urine IP-10 levels in TB patients changed over the course of treatment with a complete set of serial specimens (T0, T2, and T6). Serum IP-10 levels in TB patients at the time of diagnosis (median, 85.37 pg/ml; IQR, 60.92-171.30) were not significantly different from those after 2 months of treatment (median, 125.80 pg/ml; IQR, 81.42-155.00; *P* = 0.1743) or upon the completion of effective therapy (median, 81.09 pg/ml; IQR, 63.71-123.70; *P* = 0.7467; Fig. 2a).

However, the pairwise comparisons also showed that urine IP-10 levels in patients after 2 months of treatment (median, 19.17 pg/ml; IQR, 5.12-51.68) were significantly higher than those in patients at the time of diagnosis (median, 7.89 pg/ml; IQR, 4.86-13.97; *P* = 0.0163). Moreover, urine IP-10 levels in patients tested after the completion of drug therapy (median, 5.47 pg/ml; IQR, 4.69-9.79) were significantly lower than those sampled at the time of diagnosis (*P* = 0.0354) and after 2 months of treatment (*P* = 0.0035; Fig. 2b).

To confirm that the IP-10 response derived from TB infection, the urine IP-10 level of each patient was normalized to the serum creatinine level at each visit using test results available from routine analyses. As an abnormal serum creatinine level may indicate kidney disease or damage, this served to verify that the dynamics of IP-10 levels detected in this study were not influenced by kidney disease. The urine IP-10/creatinine ratio was higher in TB patients after 2 months of treatment than either before or after treatment, consistent with the trend observed for urine IP-10 without normalization (Fig. 2c). This indicated that the serum creatinine levels did not affect the urine IP-10 levels in this study.

### Longitudinal analysis of IP-10 levels in patients with active TB stratified by risk group

The group of TB patients was then sub-divided by risk factors for relapse or treatment failure into low, moderate, and high-risk groups. Among all patients, 15 (65.2%) were included in the low-risk group, and 8 (34.8%) were included in the moderate/high-risk group.

**Fig. 3:**
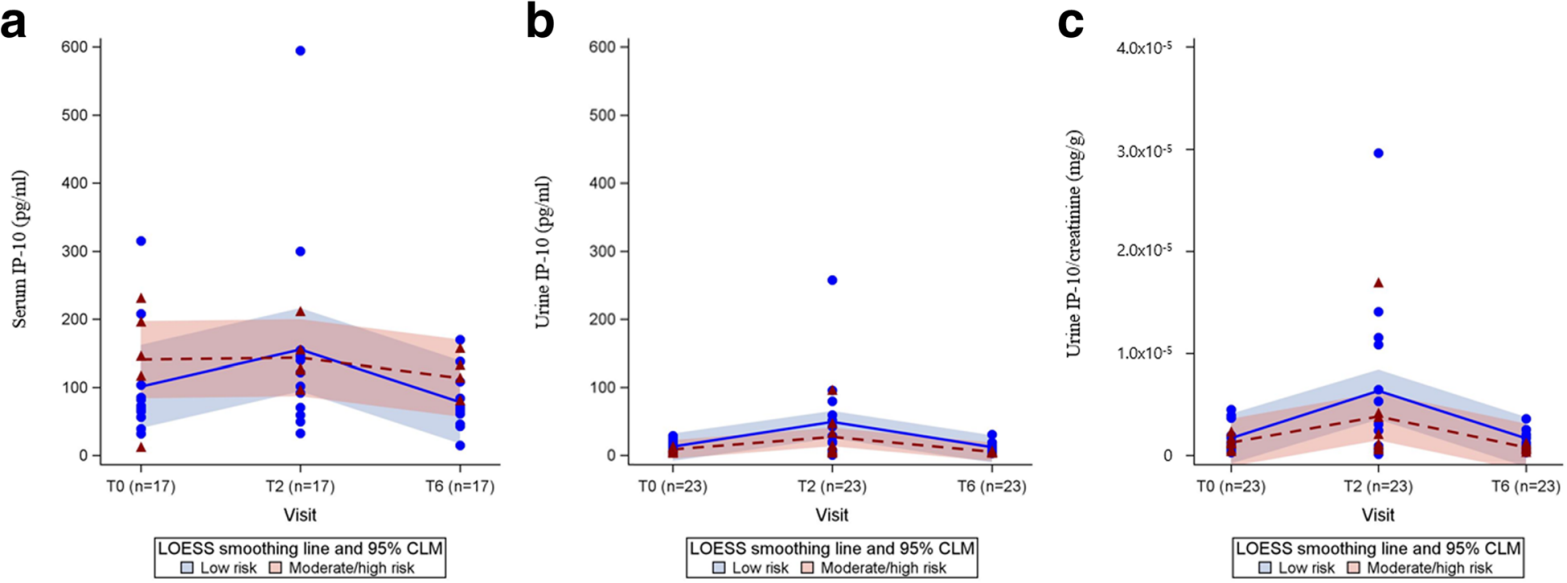
Longitudinal analysis of IP-10 levels in patients with active TB stratified by risk of relapse. Longitudinal (**a**) serum, (**b**) urine IP-10, and (**c**) urine IP-10/creatinine ratio obtained throughout treatment (T0, T2, and T6) were analysed in risk subgroups (low-risk and moderate/ high-risk groups) using locally weighted scatterplot smoothing (LOESS) and confidence intervals of the mean (CLM)

As shown in Fig. 3b and c, the smoothed lines for urine IP-10 in both risk groups exhibited a similar trend over the three time points. The low-risk group had higher urine IP-10 levels than those of the moderate/high-risk group, but the 95% confidence limits of IP-10 levels were not differentiated by risk group.

In contrast, serum IP-10 levels did not show similar trends in the two risk groups (Fig. 3a). Serum IP-10 levels in the moderate/high-risk group did not change over time, but those in the low-risk group increased at T2 and decreased at T6, similar to the results for urine IP-10 levels.

## Discussion

As sputum culture conversion at 2 months is not always predictive of successful TB therapy, new biomarkers for predicting treatment responses are needed for better management of TB patients. Similar to the findings of Cannas et al. [20], this study showed a declining tendency for urine IP-10 detection in TB patients after receiving effective anti-TB drug therapy compared the levels at time of diagnosis. However, this study also detected urine IP-10 levels after 2 months of an intensive anti-TB drug therapy, which is the point when clinicians routinely use radiological and bacteriological tests to determine treatment progress. Although recent studies have reported a decline in blood IP-10 levels after 2 weeks of efficient anti-TB drug therapy as an early biomarker for treatment response [21, 22], this study demonstrated a peak in urine IP-10 levels after 2 months of treatment in HIV-negative patients with tuberculosis. Moreover, the same trend was observed in patients regardless of risk factors for TB relapse or treatment failure.

It is not clear why unstimulated IP-10 levels in urine significantly increase in patients after 2 months of anti-TB treatment. A previous study using imaging scans suggested quantitative changes at 2 months after the initiation of treatment, which may be related to long-term TB treatment outcome [23]. To confirm this phenomenon, additional investigations with a large-scale active TB cases including prolonged anti-TB treatment (over 12 months) may be useful to ensure the timing of peak inflammation and urine IP-10 for successful treatment.

Individuals with acute and chronic infections were excluded from analyses, and urine IP-10 levels were normalized against serum creatinine levels to ensure that increases in urine IP-10 levels can be attributed to infection with *M. tuberculosis,* rather than kidney disease or damage. In future studies, urine creatinine levels should be examined to directly exclude urinary disease.

In addition, C-reactive protein (CRP) is an indicator of disease activity and is often increased in active tuberculosis inflammatory conditions [24, 25]. However, CRP data for patients at diagnosis and follow-ups were not available in this study. In future studies, CRP levels should be estimated and their correlations with IP-10 levels can be used to assess inflammatory changes in response to TB treatment. Furthermore, this study emphasizes the usefulness of urine as non-invasive sample for monitoring treatment responses biomarkers in children, the elderly and HIV-infected subjects.

## Conclusions

In conclusion, unstimulated IP-10 levels in patient urine samples, which are easier to collect and analyse than blood samples, can be used as a predictive factor, indicating a positive treatment response. IP-10 detection may therefore be a useful monitoring tool for determining whether TB patients are positively responding to anti-TB drug therapy.

## References

[1] WHO (2015). Global tuberculosis report.

[2] WHO (2016). Global tuberculosis report.

[3] Zumla A, Nahid P, Cole ST (2013). Advances in the development of new tuberculosis drugs and treatment regimens. Nat Rev Drug Discov.

[4] Walzl G, Ronacher K, Djoba Siawaya JF, Dockrell HM (2008). Biomarkers for TB treatment response: challenges and future strategies. J Inf Secur.

[5] Nahid P, Dorman SE, Alipanah N, Barry PM, Brozek JL, Cattamanchi A (2016). Executive summary: official American Thoracic Society/Centers for Disease Control and Prevention/Infectious Diseases Society of America clinical practice guidelines: treatment of drug-susceptible tuberculosis. Clin Infect Dis.

[6] Horne DJ, Royce SE, Gooze L, Narita M, Hopewell PC, Nahid P (2010). Sputum monitoring during tuberculosis treatment for predicting outcome: systematic review and meta-analysis. Lancet Infect Dis.

[7] Dembele SM, Ouedraogo HZ, Combary A, Saleri N, Macq J, Dujardin B (2007). Conversion rate at two-month follow-up of smear-positive tuberculosis patients in Burkina Faso. Int J Tuberc Lung Dis.

[8] Blumberg HM, Burman WJ, Chaisson RE, Daley CL, Etkind SE, Friedman LN (2003). American Thoracic Society/Centers for Disease Control and Prevention/Infectious Diseases Society of America: treatment of tuberculosis. Am J Respir Crit Care Med.

[9] Benator D, Bhattacharya M, Bozeman L, Burman W, Cantazaro A, Chaisson R (2002). Rifapentine and isoniazid once a week versus rifampicin and isoniazid twice a week for treatment of drug-susceptible pulmonary tuberculosis in HIV-negative patients: a randomised clinical trial. Lancet.

[10] Reid MJ, Shah NS (2009). Approaches to tuberculosis screening and diagnosis in people with HIV in resource-limited settings. Lancet Infect Dis.

[11] Getahun H, Harrington M, O’Brien R, Nunn P (2007). Diagnosis of smear-negative pulmonary tuberculosis in people with HIV infection or AIDS in resource-constrained settings: informing urgent policy changes. Lancet.

[12] Hargreaves NJ, Kadzakumanja O, Whitty CJ, Salaniponi FM, Harries AD, Squire SB (2001). Smear-negative' pulmonary tuberculosis in a DOTS programme: poor outcomes in an area of high HIV seroprevalence. Int J Tuberc Lung Dis.

[13] Goletti D, Petruccioli E, Joosten SA, Ottenhoff THMM (2016). Tuberculosis biomarkers: from diagnosis to protection. Infect Dis Rep.

[14] Wallis RS, Doherty TM, Onyebujoh P, Vahedi M, Laang H, Olesen O (2009). Biomarkers for tuberculosis disease activity, cure, and relapse. Lancet Infect Dis.

[15] Petrone L, Cannas A, Vanini V, Cuzzi G, Aloi F, Nsubuga M (2016). Blood and urine inducible protein 10 as potential markers of disease activity. Int J Tuberc Lung Dis..

[16] Profumo E, Buttari B, Tosti ME, Alessandri C, Valesini G, Marcuccio L (2010). Identification of IP-10 and IL-5 as proteins differentially expressed in human complicated and uncomplicated carotid atherosclerotic plaques. Int J Immunopathol Pharmacol.

[17] Hong JY, Lee HJ, Kim SY, Chung KS, Kim EY, Jung JY (2014). Efficacy of IP-10 as a biomarker for monitoring tuberculosis treatment. J Inf Secur.

[18] Riou C, Perez Peixoto B, Roberts L, Ronacher K, Walzl G, Manca C (2012). Effect of standard tuberculosis treatment on plasma cytokine levels in patients with active pulmonary tuberculosis. PLoS One.

[19] Kabeer BSA, Raja A, Raman B, Thangaraj S, Leportier M, Ippolita G (2011). IP-10 response to RD1 antigens might be a useful biomarker for monitoring tuberculosis therapy. BMC Infect Dis.

[20] Cannas A, Calvo L, Chiachio T, Cuzzi T, Vanini V, Lauria FN, et al. IP-10 detection in urine is associated with lung diseases. BMC Infect Dis. 2010;10 10.1186/1471-2334-10-333 PMC2995466 21092156

[21] Tonby L, Ruhwald M, Kvale D, Dyrhol-Riise AM (2015). IP-10 measured by dry plasma sopts as biomarker for therapy responses in mycobacterium tuberculosis infection. Sci Rep.

[22] Garcia-Basteiro AL, Mambuque E, den Hertog A, Saavedra B, Cuamba I, Oliveras L (2017). IP-10 kinetics in the first week of therapy are strongly associated with bacteriological confirmation of tuberculosis diagnosis in HIV-infected patients. Sci Rep.

[23] Coleman MT, Chen RY, Lee M, Lin PL, Dodd LE, Maiello P, et al. PET/CT imaging reveals a therapeutic response to oxazolidinones in macaques and humans with tubercuosis. Sci Transl Med. 2014;6:265ra167. 10.1126/scitranslmed.3009500. 10.1126/scitranslmed.3009500 PMC6413515 25473035

[24] Rohini K, Bhat MS, Srikumar PS, Mahesh KA (2016). Assessment of hematological parameters in pulmonary tuberculosis patients. Indian J Clin Biochem.

[25] Brown J, Clark K, Smith C, Hopwood J, Lynard O, Toolan M (2016). Variation in C–reactive protein response according to host and mycobacterial characteristics in active tuberculosis. BMC Infect Dis.

